# Clear cell and papillary renal cell carcinomas in hereditary papillary renal cell carcinoma (HPRCC) syndrome: a case report

**DOI:** 10.1186/s13000-021-01170-8

**Published:** 2021-11-20

**Authors:** Sophie Ferlicot, Pierre-Alexandre Just, Eva Compérat, Etienne Rouleau, Frédérique Tissier, Christophe Vaessen, Stéphane Richard

**Affiliations:** 1 Service d’Anatomie Pathologique, AP-HP, Université Paris-Saclay, Hôpital de Bicêtre, Service d’Anatomie Pathologique, 78 rue du Général Leclerc, 94270 Le Kremlin-Bicêtre, France; 2 Réseau National de Référence pour Cancers Rares de l’Adulte PREDIR labellisé par l’INCa, Hôpital de Bicêtre, AP-HP, Service d’Anatomie Pathologique, 78 rue du Général Leclerc, 94270 Le Kremlin-Bicêtre, France; 3 EPHE, PSL Université, 75014 Paris, France and CNRS UMR 9019, Gustave Roussy, Université Paris-Saclay, Villejuif, France; 4 Université de Paris, AP-HP. Centre, Service de Pathologie, Hôpital Cochin, Paris, France; 5 Service d’Anatomie Pathologique, AP-HP, Sorbonne Université, Hôpital Tenon, Paris, France; 6 Service de Génétique, Institut Gustave Roussy, Villejuif, France; 7 Laboratoire de Pathologie Praxea Diagnostics, Massy, France; 8 Service d’Urologie, CHU Pitié-Salpêtrière, AP-HP, Paris, France

**Keywords:** Renal cell carcinoma, Hereditary papillary renal cell carcinoma, MET

## Abstract

**Background:**

Hereditary papillary renal cell carcinoma (HPRCC) is a rare autosomal dominant disease characterized by the development of multiple and bilateral papillary type I renal cell carcinomas (RCC) and papillary adenomas caused by activating mutations in the *MET* proto-oncogene. Classically, distinctive histological features of RCC are described according to the familial renal cell carcinoma syndrome. To date, no clear cell RCC has been reported in HPRCC syndrome.

**Case presentation:**

We describe the case of a 51-year-old man with a germline *MET* mutation detected on peripheral blood testing, and no germline *VHL* mutation, who developed numerous papillary tumors but also unexpectedly clear cell renal cell carcinomas. During the follow-up, an adrenal metastasis was observed 7 years after the initial diagnosis corresponding to a clear cell RCC metastasis. By immunohistochemistry, clear cell tumors showed focal cytokeratin 7, moderate racemase, and diffuse and membranous CAIX expression, while papillary tumors expressed strong diffuse cytokeratin 7 and racemase without CAIX positivity. Using FISH, *VHL* deletion was observed in one of the clear cell tumors, and the metastatic clear cell tumor presented a trisomy of chromosomes 7 and 17. These last genomic alterations are usually detected in papillary RCC, highlighting the potential link between both histological subtypes of tumors and the HPRCC syndrome.

**Conclusions:**

The pathologist must be aware that the presence of a non-papillary RCC associated with numerous papillary tumors should not exclude the diagnostic suspicion of HPRCC and thus to perform a thorough genomic study.

## Background

It is now estimated that 3% of renal cell carcinomas (RCC) are linked to an inherited predisposition [1]. To date, a dozen of genes involved in autosomal dominant syndromes have been identified, the main ones being: *VHL*, *MET*, *FLCN*, *FH*, *TSC1*, *TSC2*, and *SDHB* [2]. The most frequent inherited RCC syndrome is von Hippel-Lindau disease with a birth incidence of 1/36,000 caused by germline mutations in the *VHL* tumor suppressor gene predisposing to the occurrence of clear cell RCC. Hereditary papillary RCC (HPRCC) is an extremely rare disorder with an estimated incidence of 1/500,000 [3, 4]. It is characterized by the development of multiple and bilateral papillary type I RCC and papillary adenomas caused by activating mutations in the *MET* proto-oncogene [3]. Familial RCC syndromes are often characterized by bilateral and multifocal tumors in the kidney, generally of the same histological type. Presence of various tumor histological types is a rare event, classically described for Birt-Hogg-Dubé (BHD) syndrome with chromophobe, oncocytic or both components. In this report, we describe for the first time the association between papillary tumors and clear cell RCC in a patient with HPRCC syndrome.

## Case presentation

### Clinical history

The patient, a 51-year-old man, without a familial background of RCC, underwent computed tomography for hypertension investigation that revealed bilateral and multifocal renal tumors. Initially, eight left partial renal tumorectomies were performed. After this initial surgery, an active surveillance was established for the right kidney by biannual magnetic resonance imaging (MRI) according to the recommendations of the French National Cancer Institute network PREDIR and the local multidisciplinary team meeting (no surgical indication for tumors less than 3 cm in patients with inherited predispositions to RCC except in the case of Hereditary Leiomyomatosis with Renal Cell Cancer).

Two years after initial surgery, the MRI revealed that tumors of the right kidney had grown, and a second surgery was performed on the right kidney with five renal tumorectomies. During the follow-up, the patient underwent a left adrenalectomy for RCC metastasis. Since this date, the stability of the kidney nodules allowed an annual radiological assessment and follow-up without specific therapy.

### Pathological findings

**Fig. 1:**
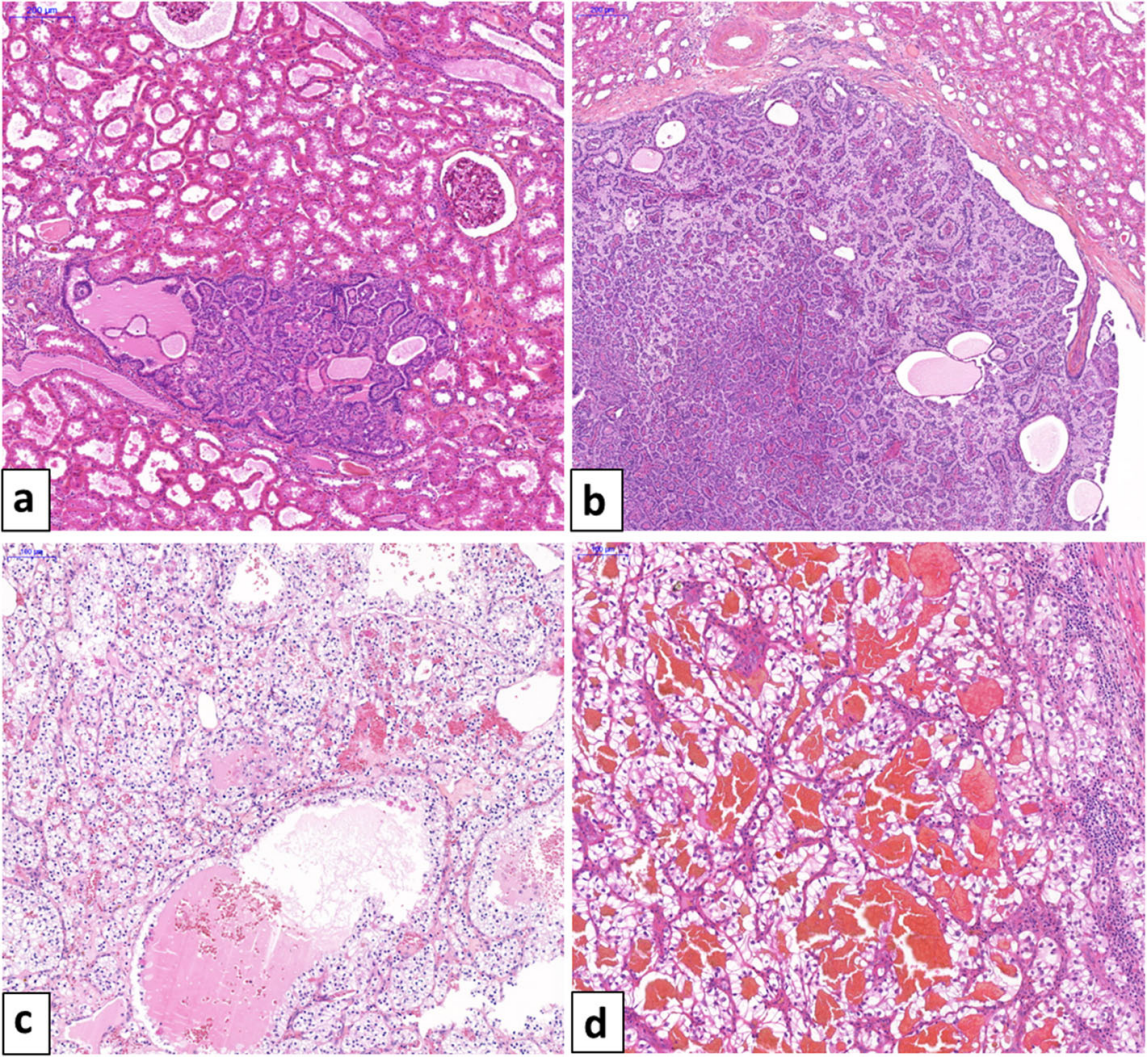
**(a)** Papillary adenoma is characterized by an encapsulated papillary proliferation arising in the renal cortex (original magnification × 50). **(b)** Papillary RCC, type 1 is characterized by papillary cores covered by a single layer of tumor cells and circumscribed by a fibrous capsule (original magnification × 50). **(c)** Clear cell RCC with alveolar nests of tumor cells and microscopic cysts (original magnification × 100). **(d)** Clear cell RCC with blood-filled microscopic cysts metastatic to adrenal gland (original magnification × 100)

The diameters of the 8 tumors of the left kidney ranged from 0.1 cm to 4 cm, and the diameters of the 5 tumors of the right kidney ranged from 0.1 to 2.5 cm. Pathological features are summarized in Table 1. Three nodules measuring respectively 2.5, 3 and 4 cm had typical histological features of clear cell RCC with ISUP grade 1 (Fig. 1c). The other nodules showed typical histological features of papillary tumors, either type 1 papillary RCC (5 tumors) or papillary adenomas often numerous (Fig. 1a and b). One of the papillary lesions (specimen n°8) was reclassified from renal cell carcinoma to adenoma after reassessment in light of the updated 2016 WHO criteria allowing papillary adenomas to be up to 1.5 cm, with no pseudocapsule [1]. Four nodules with a diameter less than 1.5 cm remained interpreted as papillary renal cell carcinomas due to encapsulation. The left adrenal nodule corresponded to a clear cell RCC metastasis (Fig. 1d).

**Table 1:** Summary of pathological, immunohistochemistry, and FISH features of the 13 renal tumorectomies and the left adrenalectomy

Specimen number	Laterality	Location	Size* (cm)	Histological type of largest tumor	Type of other tumors	P504S	CK7	CAIX	CD10	***VHL*** deletion	Trisomy 7/17
1	R	upper pole	4	clear cell RCC		++ (30%)	+ (5%)	+++ (100%)	++ (80%)	no	no
2	R	external-upper pole	3	clear cell RCC		NA	NA	NA	NA	NA	NA
3	R	posterior face	0.3	papillary adenoma		NA	NA	NA	NA	NA	NA
4	R	posterior upper pole	0.8	papillary RCC		+++ (100%)	+++ (100%)	–	–	no	yes
5	R	anterior upper pole	0.3	papillary adenoma		+++ (100%)	+++ (100%)	–	–	no	no
6	R	lower pole	1	papillary RCC		+++ (100%)	+++ (100%)	–	–	NA	NA
7	R	lower pole	0.1	papillary adenoma		+++ (100%)	+++ (100%)	–	–	no	no
8	R	upper pole	1	papillary adenoma		NA	NA	NA	NA	NA	NA
9	L	anterior face	2.5	clear cell RCC		+ (20%)	+ (5%)	+++ (100%)	++ (80%)	yes	no
10	L	anterior face	0.6	papillary RCC		NA	NA	NA	NA	no	yes
11	L	anterior face	0.1	papillary adenoma	papillary adenomas	+++ (100%)	+++ (100%)	–	+/− (5%)	no	no
12	L	upper pole	1.5	papillary RCC	papillary adenomas	NA	NA	NA	NA	NA	NA
13	L	upper pole	1	papillary RCC	papillary adenomas	+++ (100%)	+++ (100%)	–	–	no	7 only
14	L	Adrenal gland	3.1	Clear cell RCC metastasis		+ (10%)	+ (10%)	++ (90%)	++ (60%)	no	yes

*NA* not available, *RCC* renal cell carcinoma, *L* left, *R* right; * size of the largest tumor; +, mild staining; ++, moderate staining; +++, intense staining. The percentage indicated in brackets corresponds to the number of positive cells

Considering the presence of different histological types, diagnosis of BHD syndrome was initially suspected and a molecular analysis of *FLCN* gene was first performed. Absence of germline *FLCN* mutation led to realize another genetic tests targeting *VHL* and *MET* genes. Finally, on peripheral blood testing, a germline *MET* mutation c.3712G > A, p.(Val1238Ile) was found, but no *VHL* germline mutation.

An immunohistochemical analysis using TMA and fluorescent in situ hybridization (FISH) analysis were performed on ten representative nodules selected on hematoxylin and eosin-stained sections from the original blocks (2 clear cell carcinomas, 5 papillary carcinomas, 2 papillary adenomas and 1 metastasis) and are summarized in Table 1. Immunohistochemical staining was evaluated according to the percentage of stained cells and the intensity of staining as follows: +, mild; ++, moderate; and +++, intense staining. Fluorescence in situ hybridization (FISH) was performed for *VHL* loss and gain (trisomy) of chromosome 7 or 17 in the same specimens than immunohistochemical analysis. Deletion of chromosome 3p was assessed using a probe cocktail containing probes to chromosome 3p25.3 and centromere 3 (Z-2084, Zytovision). Chromosomes 7 and 17 gains were assessed using a probe cocktail containing probes to centromere 7 and centromere 17 (Z-2081, Zytovision). For each slide, 50 tumor cell nuclei were scored for probe signals under the fluorescence microscope with × 60 magnification. *VHL* loss was defined using a cut-off value of 30% of tumor cells with less 3p25.3 signals than centromere 3 signals. Gain of chromosomes 7 and 17 was defined using a cut-off value of 20% of tumor cells with ≥3 centromere 7 and/or 17 signals.

**Fig. 2:**
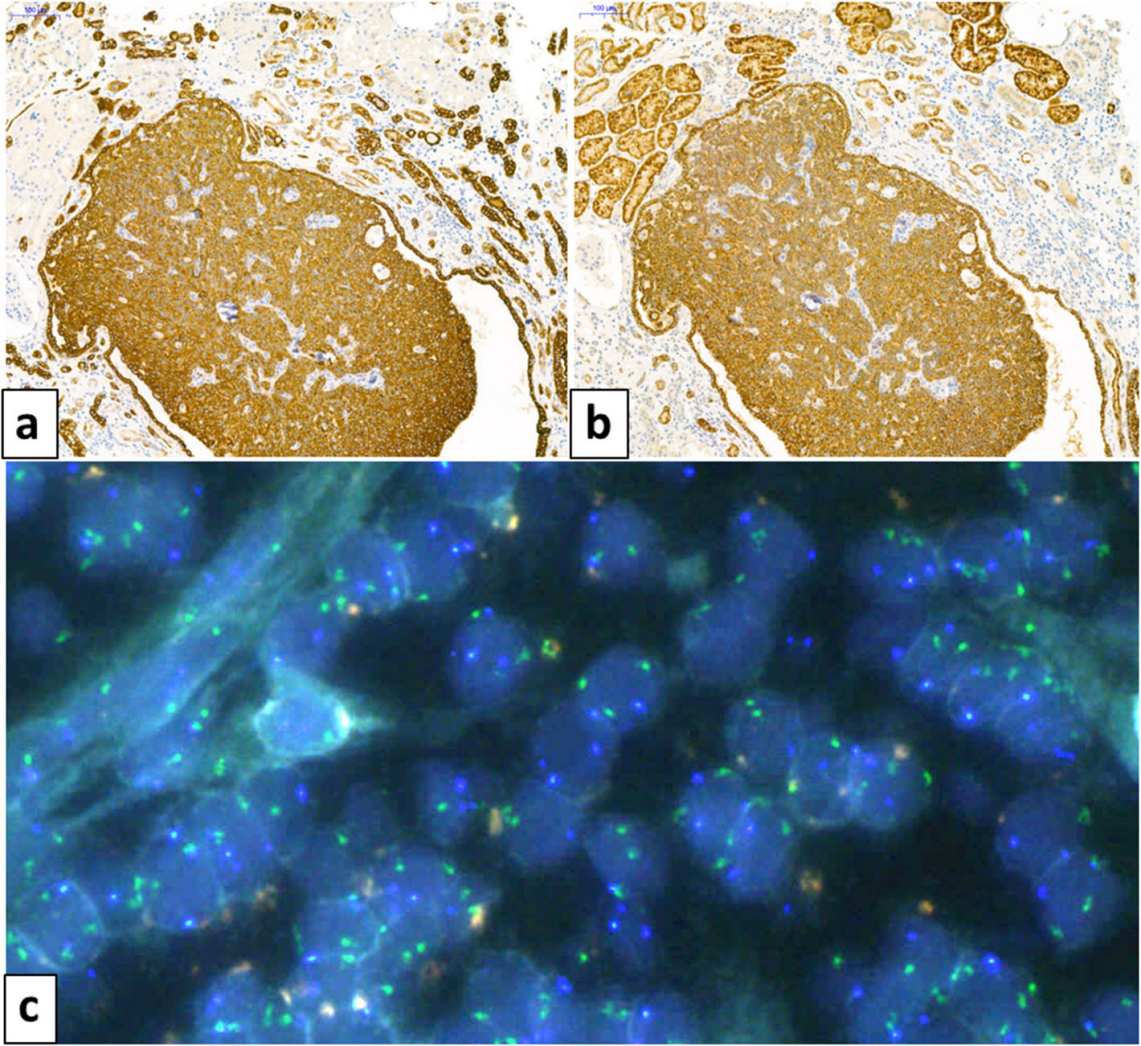
Immunohistochemical staining of a papillary adenoma showing a strong positivity for cytokeratin 7 (original magnification × 100) **(a)**, and racemase (original magnification × 100) **(b)**. Centromere 7 (green signals) and centromere 17 (blue signals) FISH evidenced a 7/17 trisomy in a papillary carcinoma **(c)**

**Fig. 3:**
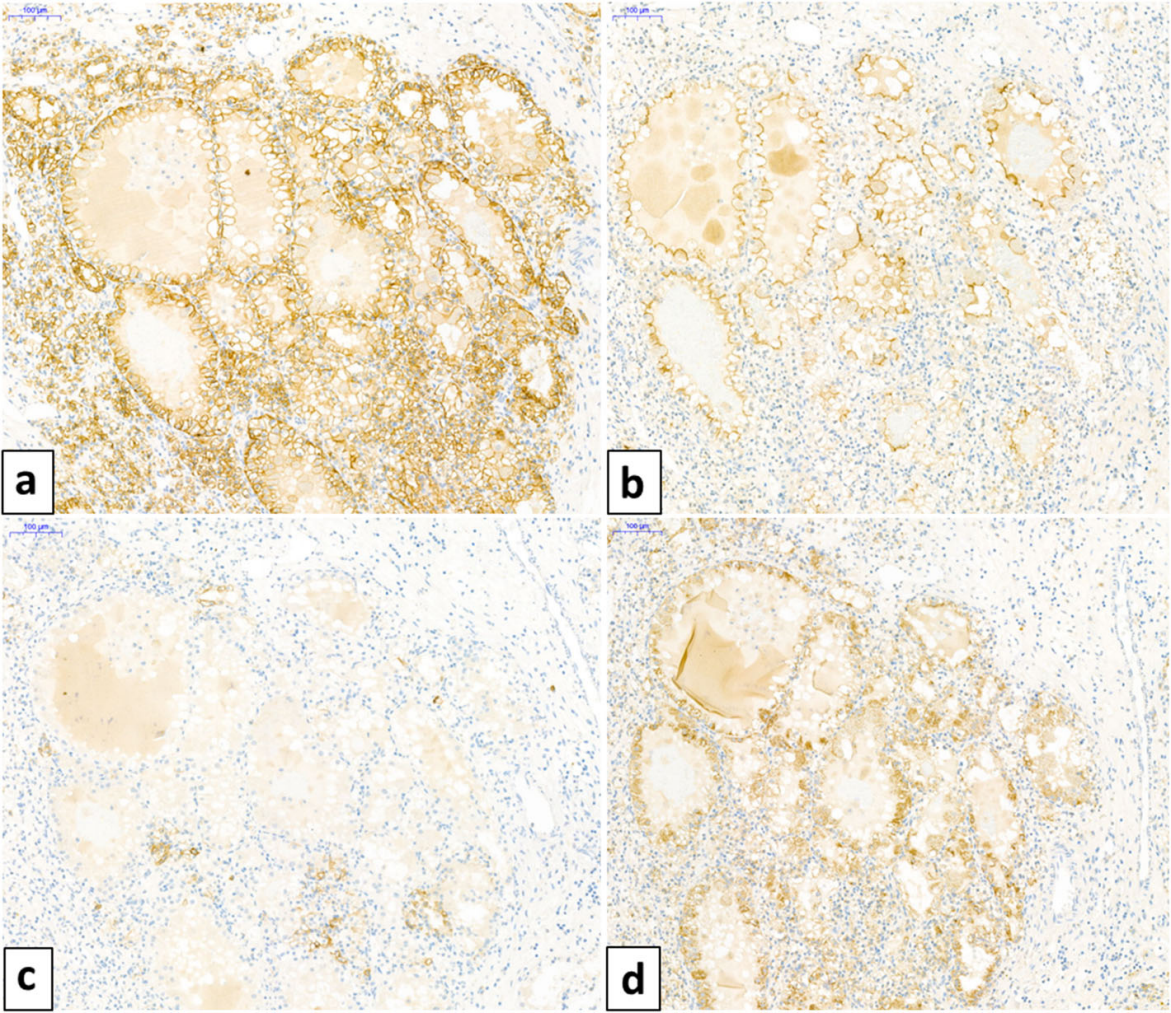
Immunohistochemical profile of a clear cell tumor (original magnification × 100). Diffuse and membranous staining with CAIX **(a)**, luminal staining with CD10 **(b)**, focal staining with CK7 **(c)**, and moderate staining with racemase **(d)**

By immunohistochemistry, all papillary lesions were positive for vimentin, cytokeratin 7 (Fig. 2a), and all had stronger labeling for alpha-methylacyl-coA-racemase (p504S) (Fig. 2b). Papillary lesions were uniformly negative for CAIX. Diffuse and membranous CAIX staining (Fig. 3a), and luminal CD10 staining (Fig. 3b) labeled only the 3 clear cell RCC. There was also a focal cytokeratin 7 (Fig. 3c) and moderate racemase (Fig. 3d) expression in clear cell tumors. Using FISH, *VHL* deletion was observed in only one case corresponding to the clear cell RCC resected in 2010. Trisomy of chromosomes 7 or 17 was encountered in 3 cases of papillary RCC and in the adrenal ccRCC metastasis (Fig. 2c).

## Discussion and conclusions

We report for the first time the occurrence of synchronous bilateral kidney tumors of different histological subtypes (clear cell RCC and papillary RCC) in a patient whom the genetic analysis showed the presence of a germline mutation in the *MET* oncogene, p.(Val1238Ile).

HPRCC is a very rare inherited syndrome characterized by the development of numerous papillary adenomas or papillary type 1 carcinomas [2, 5]. Germline mutations within the *MET* gene were first identified by a genome-wide analysis of HPRCC families then somatic *MET* mutations were also found in some sporadic papillary type 1 RCC [3, 6, 7]. The *MET* proto-oncogene is located on chromosome 7q31 and encodes the receptor for hepatocyte growth factor (HGF). HGF binding to MET results in autophosphorylation of tyrosines in the met kinase domain leading to activation of MAPKinase and PI3K-AKT signal cascades that drive effectors involved in cell proliferation, migration, and invasion [8, 9]. In cases of germline *MET* mutations, there is a ligand-independent constitutive kinase activation [3, 10]. Cytogenetic studies showed that the papillary renal carcinomas harboring *MET* mutations also had trisomy of chromosome 7, resulting from duplication of the chromosome harboring the mutated *MET* allele [11].

Classically, a specific histological type is described according to each inherited syndrome; for example, clear cell RCC in VHL disease and papillary tumors in HPRCC syndrome. However, recently, two cases of biphasic squamoid RCC (BSARCC) mixed with type 1 papillary RCC have been reported in a familial context of hereditary papillary RCC associated with *MET* mutation. Immunohistochemical features with expression in both populations of CK7, AMACR and vimentin were consistent with a link between BSARCC and type I papillary RCC [12]. Several hypotheses could be considered to explain the occurrence of various histological types: (1) common metabolic pathway but other cases of mixed histological subtypes should then have been reported; (2) specific histological type according to the type of mutation as described previously for clinical manifestations in VHL disease [13]. Against this possibility, to date, it has not been described any genotype-phenotype correlations in HPRCC. Furthermore, the patient presented one the most frequent *MET* known mutations. No report has described clear cell RCC or coexistence of other histologic subtypes in the same kidney of a patient with HPRCC except the report of a biphasic squamoid RCC; and (3) both clear cell RCC and papillary RCC are originated from proximal tubules and we can not exclude that additional genetic events of key regulatory genes may push a tumor towards a particular phenotype.

FISH analysis showed trisomy 7 and/or 17 in 3 out of 4 tested cases of papillary RCC and more unexpectedly in the clear cell RCC metastasis. This data would be in favor of a molecular link between both histological subtypes. Trisomy 7 and/or 17 were not observed in the papillary adenomas, perhaps influenced by difficulty evaluating sufficient numbers of tumor cells in microscopic papillary adenomas and a potential lower rate of these alterations in adenomas compared to papillary carcinomas. Interestingly, among the 3 clear cell RCC, *VHL* deletion was observed in only one case.

In summary, it is important to remember that some genomic alterations might be associated with synchronous bilateral kidney tumors with different histological types. The pathologist must be aware that the presence of a non-papillary RCC associated with numerous papillary adenomas or type I carcinomas should not exclude the diagnostic suspicion of HPRCC and thus to perform a thorough genomic study.

## Data Availability

All the data regarding the findings are available within the manuscript.

## Abbreviations

BHD: Birt-Hogg-Dubé
FISH: Fluorescence in situ hybridization
RCC: Renal cell carcinoma
HPRCC: Hereditary papillary renal cell carcinoma
